# The energy allocation trade-offs underlying life history traits in hypometabolic strepsirhines and other primates

**DOI:** 10.1038/s41598-021-93764-x

**Published:** 2021-07-09

**Authors:** Bruno Simmen, Luca Morino, Stéphane Blanc, Cécile Garcia

**Affiliations:** 1 UMR 7206 Eco-Anthropologie, CNRS - MNHN - Université de Paris, 1 avenue du Petit Château, 91800 Brunoy, France; 2 Parc Zoologique de Paris, MNHN - Sorbonne Universités, Paris, France; 3 Institut Pluridisciplinaire Hubert Curien, UMR CNRS Unistra 7178, 23 rue Becquerel, 67087 Strasbourg, France; 4 UMR 7206 Eco-Anthropologie, CNRS - MNHN - Paris Diderot, Musée de L’Homme, 17, place du Trocadéro, 75016 Paris, France

**Keywords:** Biological anthropology, Homeostasis, Animal physiology, Stable isotope analysis

## Abstract

Life history, brain size and energy expenditure scale with body mass in mammals but there is little conclusive evidence for a correlated evolution between life history and energy expenditure (either basal/resting or daily) independent of body mass. We addressed this question by examining the relationship between primate free-living daily energy expenditure (DEE) measured by doubly labeled water method (n = 18 species), life history variables (maximum lifespan, gestation and lactation duration, interbirth interval, litter mass, age at first reproduction), resting metabolic rate (RMR) and brain size. We also analyzed whether the hypometabolic primates of Madagascar (lemurs) make distinct energy allocation tradeoffs compared to other primates (monkeys and apes) with different life history traits and ecological constraints. None of the life-history traits correlated with DEE after controlling for body mass and phylogeny. In contrast, a regression model showed that DEE increased with increasing RMR and decreasing reproductive output (i.e., litter mass/interbirth interval) independent of body mass. Despite their low RMR and smaller brains, lemurs had an average DEE remarkably similar to that of haplorhines. The data suggest that lemurs have evolved energy strategies that maximize energy investment to survive in the unusually harsh and unpredictable environments of Madagascar at the expense of reproduction.

## Introduction

There are theoretical and empirical reasons to suggest that life history traits could have evolved in close relation to the metabolic requirements and energy input necessary to fuel the components of fitness^1,2^. From a theoretical point of view, the energy acquired from the environment allows to sustain survival, growth and reproduction. And, by analogy with demographic principles, at steady state, energy allocated to growth and reproduction (beside respiration) should match energy lost to mortality over a lifespan in each generation^3^. Therefore, a link is expected between life history traits, which evolve under selection pressures to promote fitness, and the amount and distribution of energy required to maintain these biological fundamentals. Empirically, the findings that primates spend fifty percent less energy than other eutherians with similar mass^2^ led to revisit the potential for correlated evolution of life history traits and energy needs in these mammals. Primates indeed have a slow life history among placental mammals^4,5^ and they must allocate energy to a comparatively larger brain in addition to growth, maintenance and reproduction. Accordingly, their slow pace of life likely evolved in relation to energy allocation trade-offs between survival and reproduction costs, as in other mammals, but also on their characteristically reduced energy budget.

In primates and other placental mammals, growth, maternal investment, and life expectancy are positively related to total daily energy expenditure (DEE)^4,6^ but since most life history traits and brain size are connected to body mass, e.g.,^1,7^, looking for a correlated evolution between life history and energy expenditure necessitates to remove the effect of body mass. Early studies that tackled this issue while controlling for body size and phylogeny used resting metabolic rate (RMR, i.e., the amount of energy expended by a fasting individual at rest and at thermoneutrality and measured under conditions less stringent than basal metabolic rate) as a proxy for a species’ total energy demand^8,9^. The reason for using RMR preferably over DEE (i.e., the total amount of energy spent daily by an individual for all its physiological and behavioral activities) was that there were many measurements of RMR available for meta-analyses. The wide range of species tested was an advantage, but these early studies actually failed to find a general relationship between life history and RMR^8,9^ (perhaps because the RMR does not capture the full spectrum of energy expenditure). Similarly, a study focusing on strepsirhine primates could not demonstrate a correlation between maternal reproductive investment and RMR independently from body mass and after controlling for the effect of phylogeny^10^. A recent meta-analysis on eutherian mammals, including primates^2^, found a positive relationship between daily energy expenditure and reproductive output (litter mass/interbirth interval) controlling for body mass but the link was weak due to the great variability of reproductive data and was not found for two other life history traits (growth and maximum lifespan). The lack of a consistent relationship at high taxonomic level suggested that the results may be blurred by the trade-offs in energy allocation that species or taxonomic groups make according to their reproductive cycle and lifestyle^2^. It is also possible that studies that focused on DEE in primates were inconclusive due to the limited set of life history traits they tested.

In the present paper, we examined these relations further using the set of published results on primate DEE, augmented with new data in a lemur species. We combined these measurements with an array of life history traits in simple and multiple regression models. The DEE results used here were all obtained with the doubly labeled water method, the gold standard for measuring total energy expenditure in free-living conditions. Additionally, we focused on a group of primates, the lemurs of Madagascar (Lemuriformes, Strepsirhini), that differ from other primates (Haplorhini, i.e. the New and Old World monkeys, including apes and humans) in many aspects of their socioecology, physiology and life history. Lemurs, for example, have short gestation and lactation times, small brain volume, low baseline energy requirements, and this group includes a few heterothermic species^11^. The aim was to highlight the energy allocation trade-offs in species that have evolved in Madagascar in habitats characterized by unusually harsh biotic and abiotic constraints^12–17^. Stochastic climate events in Madagascar include drought years, occasional food shortage during the expected period of food abundance during the wet months, and cyclones^16^. This climate pattern seems to have been present for the last 5 million years, which has undoubtedly had an impact on the evolution of lemur energetics^15^. In this study, we show that primate species with a high RMR but low reproductive output have high DEE for their body mass. Contrary to our prediction that energy limitation in Madagascar selected for low energy expenditure strategies, we provide evidence that lemurs do not differ from other primates on average, but invest comparatively less energy for reproduction than for somatic maintenance.

## Results

### Scaling of DEE with body mass in Malagasy lemurs and other primates

**Figure 1:**
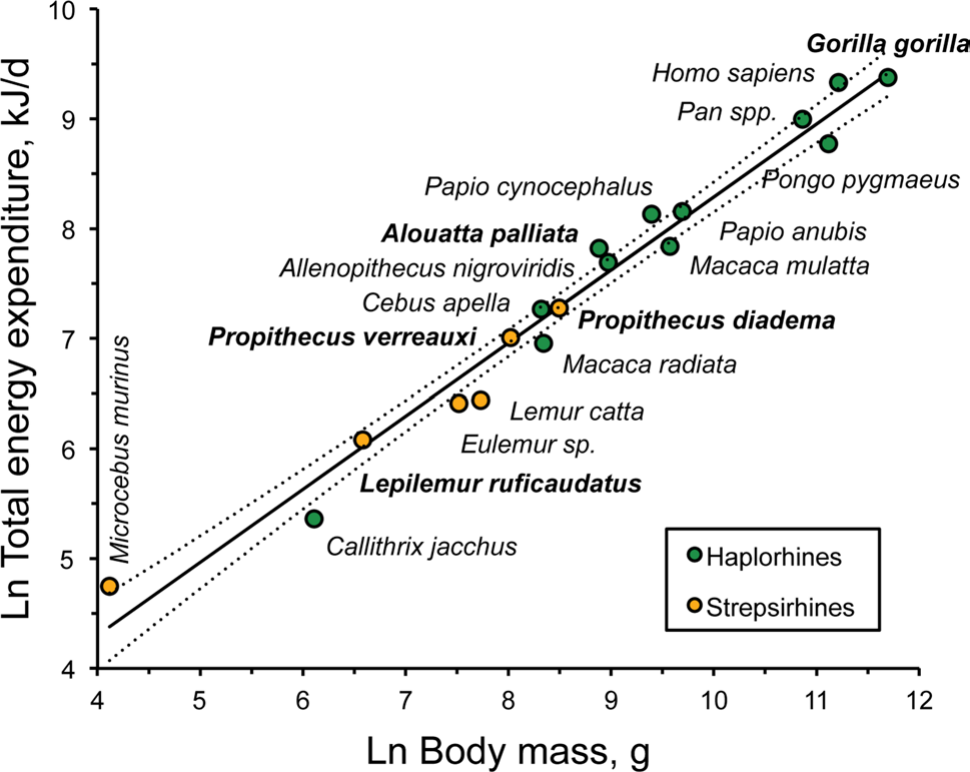
Scaling of energy expenditure with body mass among strepsirhine and haplorhine species after logarithmic transformation of the data. Species with a folivorous tendency are identified in bold. In this PGLS regression analysis, the equation is LnDEE (kJ/d) = 1.64 (± 0.24) + 0.66 (± 0.03) * LnBody mass (g), with 97% of the DEE variance explained by body mass variation.

The regression of DEE on body mass was established to extract DEE residuals in each species for later statistical analyses (see following sections) and to provide an update of DEE variation among primates including our new results on the Verreaux’s sifaka. DEE data used here were collected from non-gestating, non-lactating adults, therefore mainly reflecting basal energy requirements, physical activity, and physiological regulation for maintaining body homeostasis (Table 1). With this set of 18 human and nonhuman primate species, the phylogenetic generalized least square (PGLS) regression of DEE on species body mass (Fig. 1) conformed to the expected pattern: after Ln-Ln transformation, the slope of the regression line was 0.66, which corresponds to previous estimates and shows that the DEE per unit body mass is lower in larger species^2,18,19^.

**Table 1:** Metabolic requirements in selected strepsirhine (S) and haplorhine (H) primates.

Genus species	Clade	Body mass kg	DEE kJ d^−1^	RMR kJ d^−1^	PAL
*Eulemur fulvus*	S	1.84 w	609 w	145*	4.20
*Lemur catta*	S	2.28 w	626 w	330	1.89
*Lepilemur mustelinus*	S	na	na	na	na
*Lepilemur ruficaudatus*	S	0.721 w	438 w	220	3.65
*Microcebus murinus*	S	0.061 w	115 w	22*	5.12
*Propithecus diadema*	S	4.9 w	1446 w	na	na
*Propithecus verreauxi*	S	3.04 w	1107 w	360*	3.07
*Alouatta palliata*	H	7.22 w	2496 w	1324	1.89
*Callithrix jacchus*	H	0.45	213	112*	1.89
*Cebus apella*	H	4.1	1430	na	na
*Macaca mulatta*	H	14.4	2537	1944*	1.31
*Macaca radiata*	H	4.2	1049	na	na
*Papio anubis*	H	16.2	3478	2099	1.66
*Papio cynocephalus*	H	12.0w	3400 w	2472	1.38
*Gorilla gorilla*	H	120.0	11,829	na	na
*Pan paniscus*	H	52.15	8082	na	na
*Pan troglodytes*	H	52.15	8082	5120*	1.58
*Pongo pygmaeus*	H	67.45	6464	5088	1.27
*Homo sapiens*	H	74.25	11,300	7212*	1.57
*Allenopithecus nigroviridis*	H	7.9	2190	na	na

DEE: daily energy expenditure. RMR: resting metabolic rate adjusted for the average body mass of individuals measured in DEE studies. Prediction equation and species deviation from the RMR:mass trendline (n = 40 spp) were obtained in the present study (PGLS regression: LnRMR, in kJ.d^-1^ = 3.89 + 0.71* LnBody mass, in g). PAL: estimated physical activity level, i.e. the DEE-to-RMR ratio. *: equivalent to basal metabolic rate (BMR).. DEE data for *Pan paniscus* and *P. troglodytes* were pooled in the source paper. References: see “Methods”. w: wild.

This PGLS analysis yielded a regression coefficient (r^2^ = 0.97) similar to that resulting from an ordinary least square regression (as revealed by maximum likelihood values), and no significant phylogenetic effect was detected in the distribution of DEE (Pagel’s λ = 0). In our study and previous reports, it remains possible that the exponent is artificially inflated by the taxonomic distribution of body mass data since few haplorhines were tested in the range of low body mass while strepsirhines, with their low basal rate of oxygen consumption, are mainly small-sized species. If we exclude strepsirhines from the analysis, the exponent actually does not change markedly (0.71 ± 0.04, n = 12 haplorhines). We also noted that humans had a slightly higher relative DEE compared to great apes (Fig. 1, see also Pontzer et al.^20^ who controlled for individual adiposity). While humans have evolved unusual life history trade-offs and energy budget relative to apes (with both a large somatic and reproductive investment due to their enlarged brains, prolonged growth period, long lifespan, and short interbirth interval for their body mass^20^, they did not appear as outliers in the primate DEE:mass regression (in fact, removing humans did not modify the slope of the regression line at 0.65). Likewise, *Microcebus murinus* had a high energy expenditure relative to its body mass but, while other studies suggested a separate treatment of this heterotherm species, this small primate did not appear to be an outlier in our sample (Bonferroni outlier test).

### Comparison of DEE across taxa

**Figure 2:**
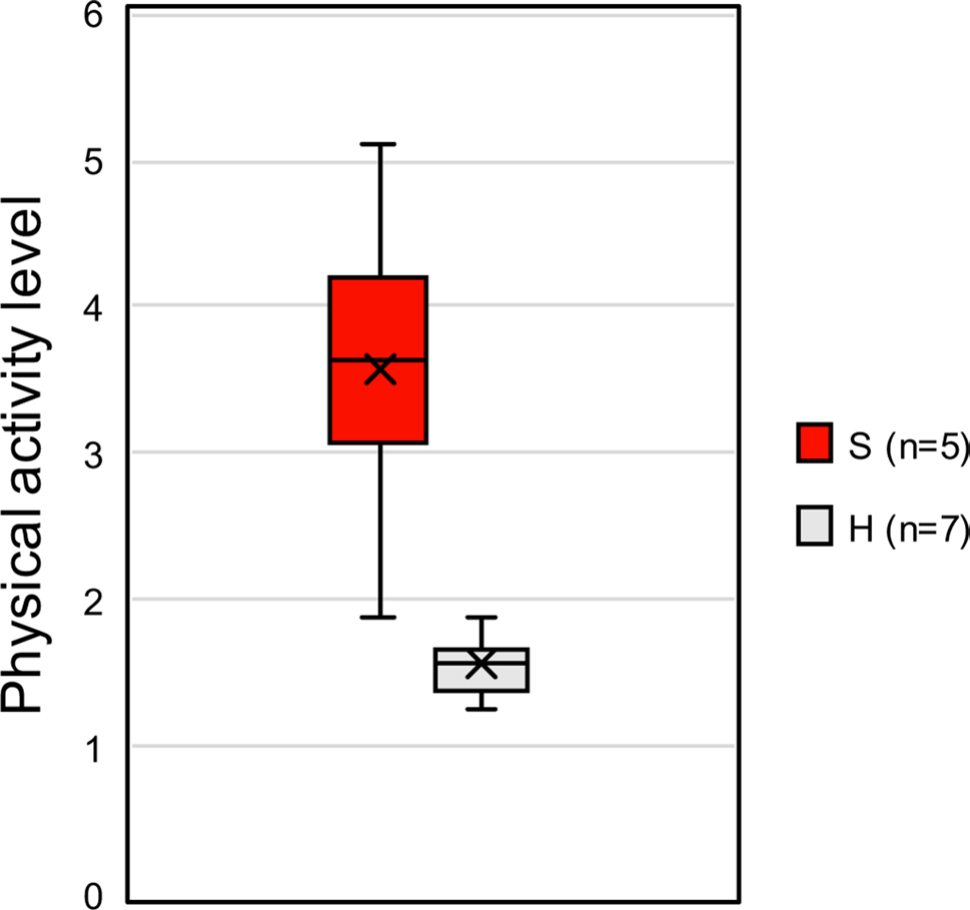
Box plot of physical activity level (DEE divided by RMR) in haplorhines (H) and strepsirhines (S). Mean value is indicated as X, close to the median (horizontal line inside the box). Upper and lower quartiles are shown as well as whiskers indicating the ranges for the bottom and top 25% of the data values.

Strepsirhines and haplorhines did not differ in their total energy expenditure after controlling for body mass (t = 0.245, *p* = 0.81, *df* 16). The lack of difference is surprising in light of the unique energy-saving mechanisms characterizing some species of lemurs tested here (e.g., Cheirogaleids: daily torpor, fat storage^21^) and the low basal metabolic rate (BMR or RMR, see below) shared by most of them^22,23^ (Table 1). The low relative RMR found in the different families of lemurs alongside the well-known small heterothermic species indeed has led to categorize lemurs as “hypometabolic” species^23,24^. As a consequence of their low metabolic needs, the DEE-to-RMR ratio (i.e., the so-called physical activity level or PAL used for humans) was higher in strepsirhines (median: 3.6) than in haplorhines (median: 1.6, W = 1, *p* < 0.01, Fig. 2). It should be noted that the DEE and RMR were not measured simultaneously on the same animals, and that PALs may be biased towards a high value in lemurs exhibiting daily torpor. However, if we remove the heterothermic lemur species *Microcebus murinus* from the analysis, the difference between the two taxonomic groups remains significant (*p* < 0.02). Given the much higher PAL of lemurs, it seems probable that the PAL difference with haplorhines is real. Accordingly, lemurs would spend a much larger portion of their total energy expenditure, on average, for sustaining the energy costs of living beyond resting requirements.

### Taxa differences in life history traits

**Figure 3:**
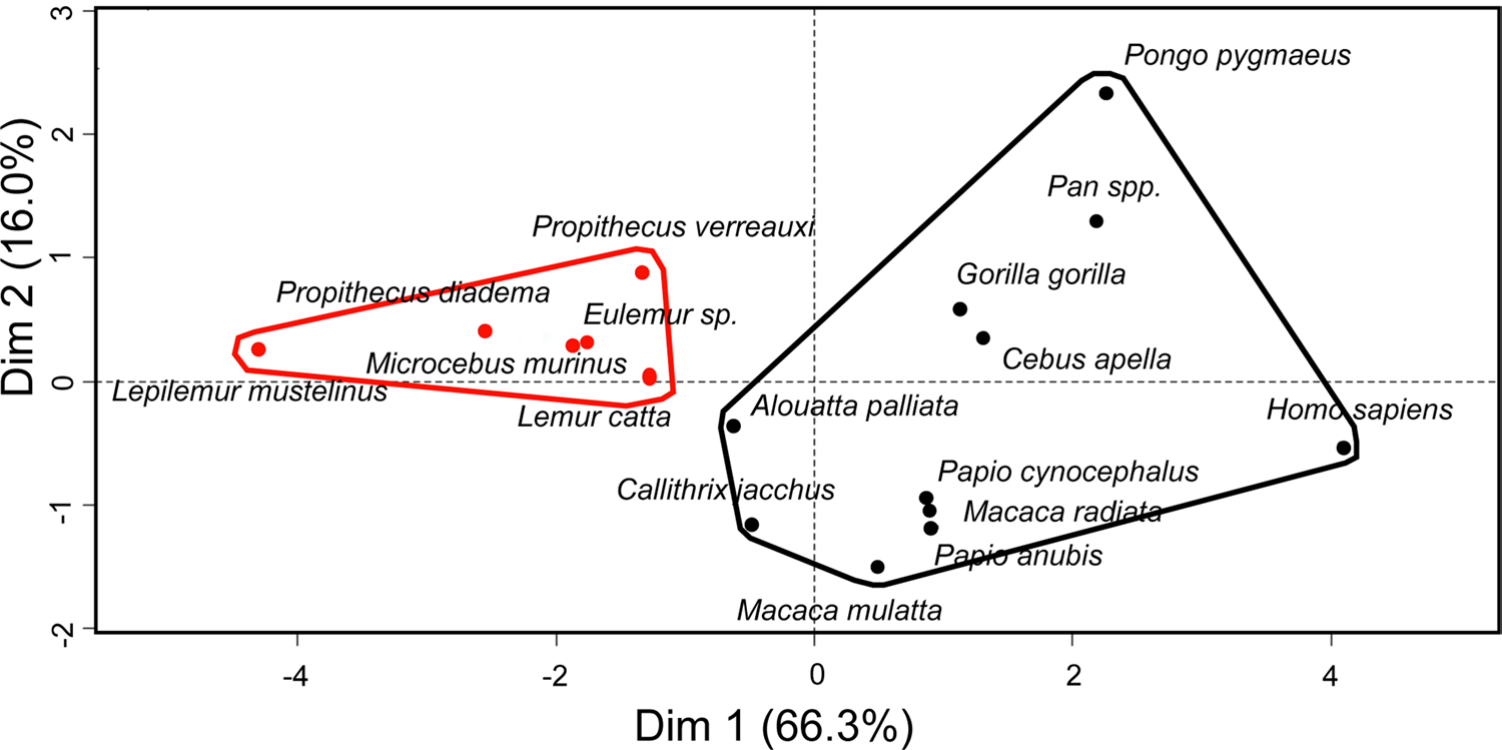
Principal component analysis showing taxonomic distinction of selected lemurs (red filled circle) from other primates (black filled circle), based on life history traits and brain size (see Table 1) when the effect of body mass is removed. Data for these species are residuals from a regression on body mass of each of the life history characteristics in a large primate sample (n = 87 spp. including humans, see Text). Dimension 1 loads positively on brain size, prenatal growth, age at first reproduction, maximum lifespan, reproductive output, and reproductive duration. Dimension 2 loads positively on reproductive duration, and negatively on reproductive output. The graph was generated using R ^64^. A biplot of the species graph and variables graph is found in Supplementary Fig. S1 online.

Maximum likelihood values used to assess the best regression model for each life history variable (Table 2) plotted against body mass in 87 primate species were indicative of a clear phylogenetic signal in all traits tested (with Pagel’s λ varying between 0.82 and 1). We found differences (Table 3) between the strepsirhines and haplorhines tested for DEE, namely differences between residuals calculated from these PGLS regressions, for the following life history traits: reproductive output (litter mass/interbirth interval), lactation duration, and reproductive duration (i.e., combined duration of gestation and lactation, see “Methods”) were significantly higher or longer in haplorhines. In contrast, no taxonomic group difference was found for maximum lifespan and age at first reproduction. On average, the relative RMR and brain volume, like history traits mentioned above, were significantly lower in strepsirhines. These taxonomic differences, visualized in a principal component analysis (Fig. 3), globally mirrored the variation previously reported with a larger sample of primate species^25,26^ and confirmed the representative nature of our sample for subsequent correlation analyses.

**Table 2:** Life history traits and brain size in selected primates.

Genus species	Endo-cranial volume cm^3^	Neonatal mass g	Litter size	Inter-birth interval d	Gestation d	Lactation d	Age at first reproduction y	Max lifespan y
*Eulemur fulvus*	24.8	78.6	1.08	547	121	183	2.66	35.5
*Lemur catta*	22.1	81.2	1.3	432	136	179	3	37.3
*Lepilemur mustelinus*	8.3	27	1	365	135	75	1.63	12
*Lepilemur ruficaudatus*	na	na	na	na	na	na	na	na
*Microcebus murinus*	1.6	7.1	1.9	365	60	40	1.75	18.2
*Propithecus diadema*	38.3	145	1	657	179	183	5.33	21
*Propithecus verreauxi*	26.2	99.7	1.17	624	159	183	6	30.5
*Alouatta palliata*	51.2	318	1.1	684	186	365	4	24
*Callithrix jacchus*	8.2	30.2	2.22	217	144	77	1.67	22.8
*Cebus apella*	64.2	208.9	1	587	152	265	6.68	46
*Macaca mulatta*	84.3	478.3	1.01	547	167	192	5	34
*Macaca radiata*	70.5	398	1.01	468	168	365	4	30
*Papio anubis*	155.3	947.5	1	757	180	420	6.92	31.6
*Papio cynocephalus*	149.5	770	1	707	175	456	5.99	34.1
*Gorilla gorilla*	433.5	2123.5	1.01	1826	257	1278	10.2	55
*Pan paniscus*	326.3	1447	1	1751	231	1094	14.2	54.5
*Pan troglodytes*	356.8	1845.5	1.1	1985	235	1460	13.25	59.4
*Pongo pygmaeus*	337.7	1968.1	1	2685	250	1936	15.7	56.3
*Homo sapiens*	1212.7	3319	1.01	1167	270	720	19.5	105
*Allenopithecus nigroviridis*	na	na	na	na	na	na	na	na

Brain size, age at first reproduction and maximal life expectancy are for females in species showing sexual dimorphism of body size. Otherwise, data are species means. Female body mass corresponding to life history variables and brain size in each species is documented in ^2^.

**Table 3:** Energy expenditure, brain size and life history compared between the haplorhines (H) and strepsirhines (S) selected after removing the effect of body mass.

Variables	t (or U)	*P*	*D*f	H vs S
DEE	0.060	0.95, ns	15	=
RMR	5.123	< 0.001	11	>
Endocranial volume	U = 60	< 0.001	14	>
Litter mass	9.269	< 0.001	14	>
Reproductive output (litter mass/interbirth interval)	5.691	< 0.001	14	>
Lactation duration	2.618	< 0.05	14	>
Reproductive duration (gestation + lactation)	2.970	< 0.05	14	>
Prenatal growth (litter mass/gestation duration)	5.939	< 0.001	14	>
Age at first reproduction	1.854	0.08, ns	14	=
Maximum lifespan	1.410	0.18, ns	14	=

Data are species residuals extracted from the PGLS regression of each variable against body mass in a large sample of non-human primates (life history and brain size: n = 86 non human primate spp., DEE: n = 17 spp., RMR: n = 40 spp.). The last column indicates whether haplorhines have similar ( =) or higher ( >) values than strepsirhines. Note that comparisons do not change when including humans.

### Life history correlates of DEE

Using simple regression models, neither life history traits nor brain volume correlated with DEE when the effect of body size and phylogeny were controlled. In contrast, 52% of the variation in residual DEE was explained in a model combining residual RMR as a principal effect and an interaction term combining residual reproductive output (litter mass/interbirth interval) and taxonomic group. RMR had a positive effect on DEE overall while strepsirhine reproductive output had a negative effect (Table 4, see Supplementary Fig. S2 online). Implementing this model with additional life history variables yielded minimal improvement of the goodness of fit, while removing RMR in any combination of these variables resulted in non-significant correlation with DEE (despite more species recruited in the analysis, see Table 1).

**Table 4:** Results of the best linear model accounting for daily energy expenditure variation. RMR and reproductive output (littermass/interbirth interval) as principal effects, with taxonomic group as an interaction term. S: strepsirhines.

Variables	ß coefficient ± SE	t	*P*
Intercept	− 0.29 ± 0.13	− 2.172	–
RMR	0.73 ± 0.22	3.257	0.011 *
Reproduction	0.06 ± 0.13	0.439	0.67
TaxonS	0.17 ± 0.22	0.805	0.44
Reproduction:TaxonS	− 0.83 ± 0.30	− 2.741	0.025 *
Adjusted r^2^	0.519	4.249^a^	0.039 *
AIC	− 42.14		

Variables: residuals extracted from the PGLS regression of each variable against body mass (both Ln-transformed) in a large sample of primate species (see “Methods”). Significant results are in bold. ^a^: F-statistics (df 4 and 8) for adj-r^2^.

## Discussion

Previous primate studies using simple regression models^2,10^ generally conclude that evolutionary changes in life history traits are relatively independent of evolutionary changes in BMR or DEE. However, the multiple regression model built here reveals a correlated evolution between reproductive output and DEE provided that RMR and taxonomic differences among primate groups are taken into account. Our results support the hypothesis of different trade-offs among energy costs allocated to maintenance and reproduction between strepsirhines and haplorhines. Compared with haplorhines, the lemurs tested invest less energy in basal functions (including a smaller brain mass) and reproduction – as proxied by a shorter relative duration of maternal investment, reduced prenatal growth and lower reproductive output.

We must stay cautious in the generalization of our results due to the relatively low number of species tested. Yet, the similarity in the average DEE of lemurs compared to other primates and high PAL in lemurs is unexpected. Because lemurs live in harsh habitats compared to their mainland counterparts, we predicted that ecological constraints of the island of Madagascar have selected for low energy expenditure strategies. Lemurs indeed cope with unpredictable inter-annual and intra-seasonal variations in rainfall, superimposed on a predictable but marked seasonality (including a long dry season with low food supply). Moreover, lemurs live in energy-poor habitats where the concentration of protein and lipid in fruits is low compared to, for example, fruits available to neotropical primates^17^. Even more intriguing, lemurs that exhibited a high-energy throughput strategy for their body mass were those with low quality diets (the folivorous *Propithecus* spp., *Lepilemur ruficaudatus*) or with unique physiological adaptations for saving energy during periods of stress, e.g., daily torpor in *Microcebus*. How then can we understand the lack of frugality in energy expenditure for species living in a highly challenging environment?

A first hypothesis could be that, with both a low BMR and a high ability to spend considerable energy above basal requirements, these lemurs have the potential to shift from a low- to a high-energy expenditure strategy according to the erratic environmental conditions prevailing at Madagascar. Potential variation of their energy balance is reflected in some behavioral proxies: during the austral dry winter period, the activity is reduced, the nycthemeral activity cycle is modified in cathemeral species, energy intake is low due to a diet including low-quality and scarce resources and individuals are lean, e.g.,^27–31^. During periods of food abundance, daily travelled distances increase in many species together with other energy-costly behaviors such as female targeted aggression and competition or territorial defense^16,32,33^. Even though their reproductive and hormonal cycles are synchronized by seasonal daylength variation, lemurs are not totally dependent upon exogenous abiotic cues. They show flexibility in their metabolic response to dietary and climatic changes and can adjust their physiology to cope with thermal variations and unpredictable crop failure, or capitalize on high levels of food abundance, whether in fat gain or in postponing the transition to a winter thrifty phenotype^12,28,31,34^. Our prediction of a seasonal variation of DEE, however, runs counter to the claim that mammal DEE only varies within a narrow physiological range^35,36^. This claim is based on the observation that no notable DEE differences were found between captive and wild animals from the same species, nor between seasons (as observed in a few species) nor, in human, between hunter-gatherers and sedentary human populations. To date, the only primate for which repeated isotopic measures have been published is the grey mouse lemur (*Microcebus murinus*) in Southern Madagascar. During the dry season, when ambient temperatures drop markedly, two types of individuals co-occur within the same population: individuals that stay normothermic and individuals that decrease their internal temperature and enter daily torpors. Astonishingly, little DEE differences were found between these phenotypes, nor did normothermic animals differ substantially in their DEE between the rainy season and the dry season (after controlling for body mass variation^37^). The authors suggest that the lack of seasonal variation could indicate that a decrease in the resting metabolic rate may compensate for the elevated costs of thermoregulation during the cold winter. Likewise, sportive lemurs (*Lepilemur)* living in the dry South of Madagascar seem to be able to maintain their total energy expenditure around some sort of "set point" but to vary seasonally the proportion of energy allocated to their behavioral and physiological activities^38^. The question of DEE variation in primates remains open however since substantial DEE differences independent of body size have been reported between *Microcebus* populations living in a rain forest vs a dry forest^39^.

Beside body mass and basal rate of oxygen consumption, several other major factors contributing to total energy expenditure may account for the different amount of energy expended for everyday life between haplorhines and strepsirhines of Madagascar. These are thermoregulation, immune system, parasite load, digestion costs, locomotion, sociality, etc. In the absence of a consistent set of data to compare, we will only examine shortly some of them. First, the costs of thermogenesis may be high in lemurs because thermoregulation costs increase markedly as body size decreases^40^, with lemurs spanning a range of relatively low body mass across primates. Second, in our dataset, several lemur species with high-energy expenditure (*Propithecus spp.* and *Lepilemur ruficaudatus*) undergo costly physical activities related to locomotion as compared with sympatric lemurs (*Eulemur* and *Lemur catta*) showing considerably lower energy throughput (Fig. 1). In the former species, vertical clinging and leaping is the main motion type while in the latter, quadrupedal motion types and semi-terrestriality prevail (*Eulemur* was found to be much more terrestrial during the study than usually reported). Interestingly, DEE measurements in these 4 lemur species were collected during the same phase of their reproductive cycle (the premating period) and, in *Lemur catta*, *Eulemur* and *Propithecus verreauxi*, at the same study site. Since these species share an extremely low rate of basal metabolism and were measured during non-reproductive phases, their distinct energy expenditure may primarily reflect differences in lifestyle and locomotor activity. Hoppers and leapers are hypermuscular in proportion to their total body mass^41^ and leaping is an energy-costly mode of locomotion relative to quadrupedal walking, at least at low speed or when using flexible substrates at landing^42–44^. Terrestrial quadruped primates like chimpanzees were estimated to devote only 10% of their DEE to locomotion in free-ranging conditions^45^. Since not all leapers are hypermuscular (e.g., the galagos from Africa, Lorisiforms, Strepsirhini^41^), the locomotion hypothesis needs to consider more energetic, kinematic and morphological studies. Finally, some species deviate from the DEE:body mass regression line in an unexpected way given their folivorous diet and low foraging activity. While folivory has long been associated with low energy input/low foraging effort strategies^46,47^, folivorous primates in our sample (strepsirhines: *Propithecus diadema* and *P. verreauxi*, *Lepilemur ruficaudatus*, haplorhines: *Alouatta palliata*, *Gorilla gorilla*) exhibited a high-energy throughput for their mass or spent as much or more energy than species feeding on ripe fruits and other high-quality foods. In comparison to fruit specialists, primates with digestive capacities adapted to feeding on vegetative, fibrous plant parts like mature leaves, harvest these relatively ubiquitous food resources within small home ranges, travel short distances daily, and tend to possess smaller relative brains^48–51^. A low-energy throughput strategy may also be typified by marmosets (*Callithrix jacchus*, Fig. 1): these callithrichids are not folivores but they primarily forage for plant exudates within very small home ranges, gouging tree barks with their specialized dentition and, similar to foliage digestion, using endogenous microorganisms to ferment the ß-linked polysaccharides of gums. The issue of why several folivorous species, some with a low RMR, spend as much energy on a daily basis remains unclear, especially since dietary characteristics may be confounded by the leaping mode of locomotion in some species. In our sample, species that are more frugivorous and with a DEE below expectation (e.g., *Lemur catta*, *Eulemur* sp.) have substantial body fat (^31^, see also orangutans^18^). This suggests that part of the energy they acquire could be saved as stored energy rather than being invested in costly daily activities.

In conclusion, the data suggest that while lemur DEE does not differ from that of other primates on average, a taxonomic distinction emerges on the lemurs’ distinct tradeoffs between reproduction and survival. And, among lemurs, some have evolved energy strategies and lifestyles that maximize energy investment in daily activities (*Propithecus verreauxi * and *P. diadema*, *Lepilemur ruficaudatus*) while others (e.g., *Lemur catta* and *Eulemur sp.*), with reduced energy expenditure, seem to favor strategies that allocate energy to the constitution of fat reserves before the breeding period. Fat stores cannot fully compensate for the energetic load of the mating and gestation season^30^, but they may contribute to the seemingly higher reproductive output of these species compared to lemurs with high DEE (i.e., they are closer to haplorhine reproductive output in the allometric regression). These elements converge to suggest that, even with energy-saving mechanisms, the pool of energy needed to fuel basic functions such as foraging, socializing, and surviving in the forest environments of Madagascar is unusually high, which in turn may limit the amount of energy invested in reproduction. We note in this respect that infant mortality is exceptionally high in lemurs and that bet-hedging reproductive strategies occur in several species^13,52^. The proposed effect of ecological and climatic constraints in shaping the energy allocation of lemurs may also explain why these taxa are not able to derive sufficient energy for growing costly tissues, especially the brain which requires constant and substantial energy supply throughout the year (e.g.,^1,53^). Finally, the results lead to a reconsideration of the nutritional constraints exerted on folivores, because (at least) some need a high supply of energy to fuel the costs of living. In particular, susceptibility to food stress and the risk of energy imbalance appears to be more critical than previously thought in at least some of these species (see also^51^). A better understanding of the causes of variation in energy balance should help trace how environmental characteristics may have shaped the evolution of life history among primates, including the large-brained humans with their distinctive suite of life history traits.

## Methods

### Primate metabolic data and life history

Total daily energy expenditure reported here has been measured with the doubly labeled water method. This isotopic method allows to determine individual active daily metabolic rate in various habitat conditions, and to make interspecific comparisons based on extremely precise energy data^6,35^. Species DEE (Table 1) were obtained from studies that focused on non-gestating, non-lactating adults (review in^2,19^) supplemented with^20,54^ and new data collection (*Propithecus verreauxi*, this study; data for humans in^55^). Results obtained for growing individuals were discarded as the rate of energy use is known to be high compared with adults. DEE data are currently available for 18 primate species (in fact, 19 species were measured but the results for chimpanzees and bonobos were pooled in the original paper), including 6 strepsirhines and 12 haplorhines (New World and Old World monkeys, with apes and humans, Table 1). The human DEE sample was large and included populations with low or middle income and populations with high human development index^55^. Details on the sample and context for each primate species can be found in the original publications. Verreaux’s sifakas (16 males, 9 females) were studied during the rainy season before the onset of the mating season in a gallery forest in Berenty reserve, southern Madagascar. In the present sample, strepsirhines were represented by Lemuriforms (e.g., Malagasy lemurs) that spanned almost the full range of body mass among extant strepsirhines (from the small mouse lemurs to the large Indriids). Lemurs show distinct feeding ecology (insectivorous, folivorous or frugivorous diets), reproductive and social system (gregarious multi-males multi-females and solitary species with promiscuous mating system) and lifestyle (nocturnal/diurnal, vertical clinger and leapers, arboreal and semi-terrestrial quadrupeds). The haplorhine subsample captured a large socio-ecological diversity as well. Resting metabolic rate (RMR) used to calculate species physical activity level (or PAL, i.e., the DEE-to-RMR ratio) included both resting and basal oxygen consumption data. Basal metabolic rate, which is measured on fasting individuals at thermoneutrality during the inactive period of their inactive circadian phase, was insufficiently documented in primates to be used exclusively in our analysis. However, the resting or basal values used to calculate species’ PAL were roughly balanced between the strepsirhines and haplorhines tested, minimizing a possible bias when comparing the mean PAL between these two groups (Table 1). Metabolic measurements were obtained from^20,23,56^ and^57^ for humans, totaling 40 primate species.

Life history data and associated female body mass were obtained from van Schaik and Isler ^1^ who reviewed age at first reproduction, gestation length, lactation length, litter mass, interbirth interval, maximum life expectancy and brain size in 87 primate species. From these traits, we established composite variables such as reproductive output (litter mass/interbirth interval), prenatal growth (litter mass/gestation duration) and reproductive duration (gestation + lactation duration), which variously reflected maternal energy investment. In one species where DEE was available but life history was not documented (*Lepilemur ruficaudatus*), we used life history data from a closely related species (*L. mustelinus*) with similar body mass (Table 2). Since body mass is one of the main correlates of the variation of life history and DEE, we assumed that any differences between two congeneric species of the same size would be outweighed, in our sample, by differences between species ranging from the tiny *Microcebus* to the gorilla.

### DEE measurements

A description of the doubly labeled water method, adapted from^58^ and^59^, to measure CO_2_ production can be found elsewhere for primates ^2,31^. The protocol of injection of water, sampling, storage and treatment of blood before IRMS analysis as well as calculation of DEE for *Propithecus verreauxi* are described in details in^31^. Analyses were here performed on a TC/EA elemental analyzer (Thermo Finnigan) coupled to a Delta V IRMS (Thermo Finnigan) as described in^60^.

### Data analyses

In analyses relating metabolism to life history traits, studies commonly focus on the female parent to take into account costs (or proxies) of reproduction, beyond growth and maintenance. However, since studies generally do not provide individual DEE results allowing to extract female energy data (contrary to life history traits where female characteristics are well documented), it is assumed that the sexes show a similar deviation from the DEE: mass regression line (see also^2^). Studies available in non-human primates report limited or no sex differences in DEE, even in dimorphic species, after controlling for body mass, female reproductive status or fat mass proportions^18,20,54,61^ (this study: *Propithecus verreauxi*). Some sex differences appear in grey mouse lemurs according to daily torpor use, but normothermic males and females have similar DEE^37^. It should also be noted that the DEE for a given species is most often measured on paired individuals from same social groups or individuals living under the same environmental conditions, therefore reducing potential sources of variability.

To establish the allometric relationships between DEE, BMR, brain size and life history on the one hand and body mass on the other hand, we used phylogenetic generalized least square (PGLS) regressions which control for phylogenetic relatedness. Indeed, non-independence of the data affects the true relationship between variables tested and may obscure their potential co-evolution. This method estimates the phylogenetic signal (λ) carried by the structure of the data set, approximated by a value which fully reflects the phylogenetic relationships (λ = 1) or on the contrary the independence of the data (λ = 0), or which is intermediate. PGLS linear regressions were performed with BayesTraits (on-line version2 of 2014^62^, using a consensus tree built from the 10ktrees website ^63^ (Genbank database for primates, version 3). Data were Ln-transformed on both axes, except for gestation duration where body mass only was Ln-transformed following Box Cox procedure.

Correlated evolution between life history traits and DEE was examined from the 18 species sampled removing the effect of body mass. Since many of these variables are inter-correlated and all are related to body mass^10^, the links between DEE and life history were assessed using, for each of these species, its residual calculated from the different PGLS regressions: the residuals from the DEE: mass regression (n = 18 spp.) on the one hand and the residuals from the life history traits: mass regression lines established using the extensive primate database (n = 87 spp.) on the other hand. Residuals were also used for comparing strepsirhines and haplorhines sampled in our DEE study with a two-tailed Student *t*-test (or Mann–Whitney *U*-test when assumptions of normality and variance homogeneity in the data were not met according to Shapiro–Wilk test and Levene-test). In all tests, the threshold for significance was set at  α ≤ 0.05. A principal component analysis was performed from residuals of the regressions on body mass of each of the life history characteristics selected. Simple and multiple linear regression models were applied with residual DEE as the response variable and residual life history traits, brain size or RMR as predictors after Ln-transforming the data to reduce heteroscedasticity. We included an interaction term *taxonomic group x life history trait* (when the latter differed significantly between strepsirhines and haplorhines) in multiple regression tests. The linear model assumptions and other validations of the models (residuals normality and homoscedasticity, autocorrelation) were assessed using the *gvlma* package and basic packages in R version 4.0.3^64^ and RStudio version 1.3.1056^65^. Principal component analysis of life history traits was performed using the *FactoMineR*, *facto extra* and *corrplot* packages in R^64^.

### Ethics statement

Experiments with *Propithecus verreauxi* complied with the European legal requirements on animal welfare (Directive 2010/63/UE) and French legislation for the ethical treatment of primates (Code rural et de la pêche maritime - art. R214-90, Décret no 2013-118 du 1er février 2013) and with the ARRIVE guidelines regarding the principles of replacement, reduction and refinement. All manipulations and treatments followed the International guidelines on health monitoring of non-human primates by FELASA (Federation of European Laboratory Animal Sciences Associations) and were approved by the scientific commitees of The Museum National d’Histoire Naturelle and Sorbonne Université (No SU-14-R-CDV-09-1). The Ministère de l’Environnement, de l’Ecologie et des Forêts of Madagascar delivered the permit to capture animals, collect and export biological samples (N°261/14/MEEF/SG/DGF/DCB.SAP/SCB). The export/import of biological samples was approved by CITES (No 732C-EA12/MG14) and by the Ministère de l’Agriculture, France (Direction Départementale de la Protection des Populations, Paris, No 75-2014-315-01).

## Supplementary Information

Supplementary Information.
