# Supplementary material for: The energy allocation trade-offs underlying life history traits in hypometabolic strepsirhines and other primates
Source: Sci Rep. 2021 Jul 9;11:14196. doi: 10.1038/s41598-021-93764-x (PMC8270931; doi:10.1038/s41598-021-93764-x)

**The energy allocation trade-offs underlying life history traits in hypometabolic strepsirhines and other primates**

Bruno Simmen, Luca Morino, Stéphane Blanc and Cécile Garcia

Supplementary Figure S1. Plot of the principal component analysis of life history traits, including the species graph and the variables graph. Life history variables are included after removing the effects of body mass and phylogeny (see text). The concentration ellipses around haplorrhines (H) and strepsirrhines (S) are shown. The graph was generated using R [64].


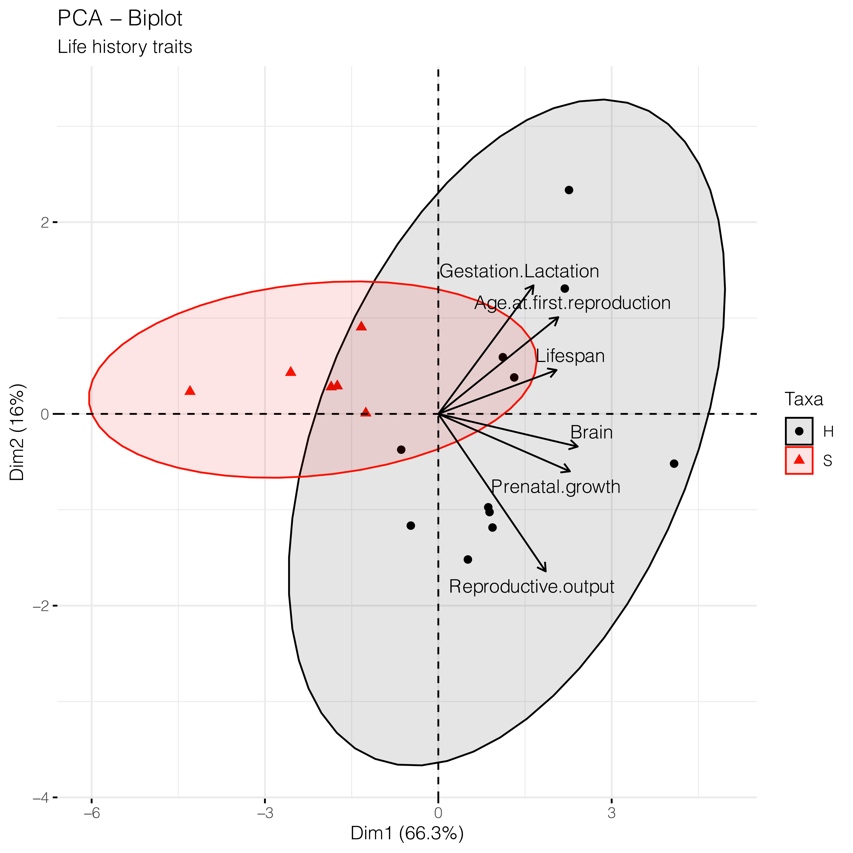


Supplementary Figure S2. Predictor effect graph derived from modelling total energy expenditure (TEE) versus resting metabolic rate (left) and reproductive output (littermass/interbirth interval; right) in haplorrhines (H) and strepsirhine (S) primates. The graphs were generated using R [64].


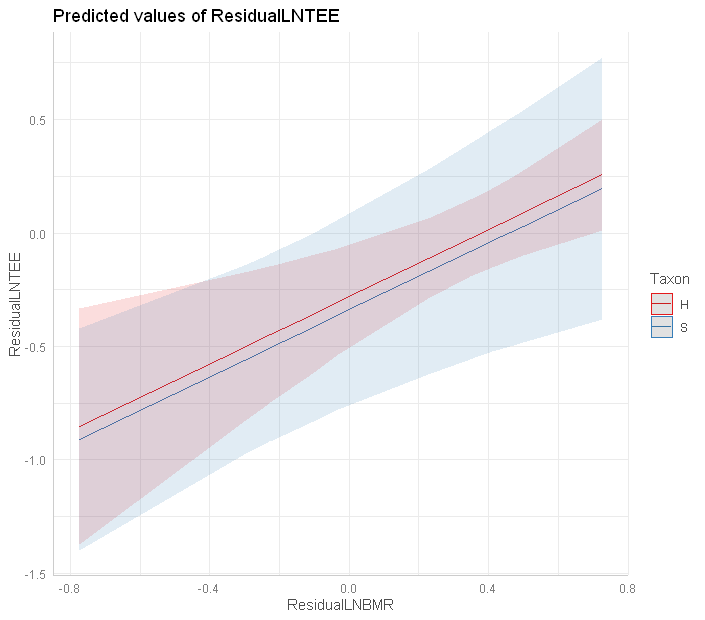

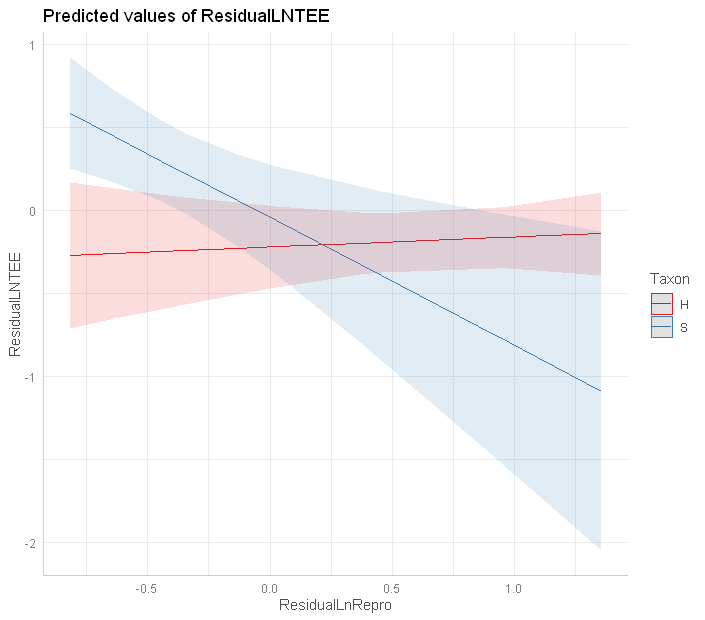

Supplement: Supplementary file 1 — Supplementary Information. [file 41598_2021_93764_MOESM1_ESM.docx]
